# Preparation of core–shell structured CaCO_3_ microspheres as rapid and recyclable adsorbent for anionic dyes

**DOI:** 10.1098/rsos.170697

**Published:** 2017-09-06

**Authors:** Mengen Zhao, Zhenhua Chen, Xinyan Lv, Kang Zhou, Jie Zhang, Xiaohan Tian, Xiuli Ren, Xifan Mei

**Affiliations:** Jinzhou Medical University, Jinzhou 121001, People's Republic of China

**Keywords:** core–shell, CaCO_3_ microspheres, dye adsorption, anionic dyes, recycling capability

## Abstract

Core–shell structured CaCO_3_ microspheres (MSs) were prepared by a facile, one-pot method at room temperature. The adsorbent dosage and adsorption time of the obtained CaCO_3_ MSs were investigated. The results suggest that these CaCO_3_ MSs can rapidly and efficiently remove 99–100% of anionic dyes within the first 2 min. The obtained CaCO_3_ MSs have a high Brunauer–Emmett–Teller surface area (211.77 m^2^ g^−1^). In addition, the maximum adsorption capacity of the obtained CaCO_3_ MSs towards Congo red was 99.6 mg g^−1^. We also found that the core–shell structured CaCO_3_ MSs have a high recycling capability for removing dyes from water. Our results demonstrate that the prepared core–shell structured CaCO_3_ MSs can be used as an ideal, rapid, efficient and recyclable adsorbent to remove dyes from aqueous solution.

## 1. Introduction

In recent years, dyes have been widely used in textiles, plastics, paper, cosmetics, pulp manufacture, tanning, pharmaceuticals and food processing industries [1,2]. However, these dyes will pollute water resources, influence the photosynthesis of underwater plants and the growth of aquatic animals, and may even bring toxicity, carcinogenicity and teratogenicity to human beings [3–7]. The disposal of toxic effluents without proper treatment is the major source of water pollution. Therefore, the removal of dyes from industrial effluents is an urgent need for the protection of water resources [8–11]. Currently, many treatment techniques have been adopted to remove dyes from waste water, including coagulation [12], flocculation [13], oxidation [14], photo-degradation [15] and electrochemical techniques [2]. However, those methods still have certain disadvantages, such as high cost and complex operation or the need of additional catalysts [16]. Compared with the methods mentioned above, the adsorption strategy is simple, convenient and effective for removing dyes from waste water [16]. To date, a variety of materials have been developed as adsorbents, such as polymer, zeolite, clay, carbon nanotubes and activated carbon [17]. However, these adsorbents still have some challenges, such as high adsorption efficiency, easy separation and recyclability [18]. After the adsorption process, some adsorbents still have to add a desorbent or be soaked in an acid or alkaline solution to be desorbed [19]. This might lead to secondary pollution. Thus, it is still an urgent challenge to develop economic, rapid, recyclable and easily separated adsorbents.

Calcium carbonate is a low-cost and abundant material in nature [20]. CaCO_3_ microspheres (MSs) can be easily separated from the liquid phase through a simple sedimentation method in a short time, benefiting from their high density and micrometre size; thus, they can be ideally used in dye adsorption according to the solid–liquid separation property [21]. Core–shell materials possess large specific surface area and many vacant sites, which have been used a lot as adsorbents in water treatment [22,23]. Besides, to burn off the adsorbed dyes, core–shell materials are more stable and easier to maintain shape than hollow-structure materials. This thermal stability property makes core–shell structured CaCO_3_ MSs suitable reusable adsorbents. In this paper, we propose a strategy of preparing core–shell structured CaCO_3_ MSs as economical and recyclable adsorbents to remove organic dyes from water.

**Scheme 1.**
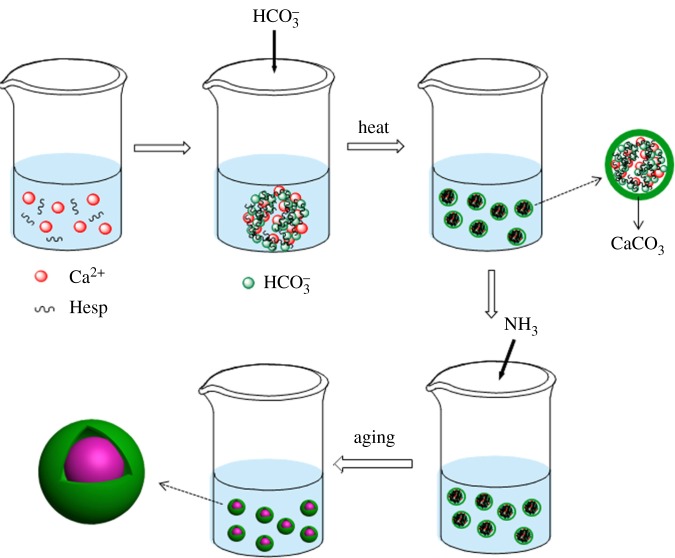
The preparation process of the core–shell CaCO_3_ microspheres.

We use a facile, one-pot method to synthesize core–shell structured CaCO_3_ MSs in the presence of hesperidin (Hesp). Hesp has been reported to regulate the formation of CaCO_3_ microspheres in our former work [24]; however, in this paper, we made a modification of the carbonate source from (NH_4_)_2_CO_3_ to NaHCO_3_. This change can provide the advantage of producing a core–shell structure in the products. The detailed steps are illustrated in scheme 1. Firstly, our protocol was to make an aqueous solution of Ca^2+^ and Hesp, which will form Ca–Hesp complexes due to the interactions of the Ca^2+^ and –OH groups on Hesp molecules [24]. Subsequently, NaHCO_3_ solution is added into the aqueous solution drop by drop. Then, ${\text{HCO}}_{3}^{-}$ is attracted by Ca^2+^ from Ca–Hesp complexes, which provide sites for further nucleation. When heating the reaction system, the outer layer of the ${\text{HCO}}_{3}^{-}$-Ca–Hesp complexes is converted into CaCO_3_ (as the shell of CaCO_3_ MSs) because of the instability of ${\text{HCO}}_{3}^{-}$ at higher temperatures. Then, NH_3_ diffusing into the system reacts with ${\text{HCO}}_{3}^{-}$, and the inner part of the complexes converts into the core of CaCO_3_ MSs. Finally, after aging for a certain time, the core–shell structured CaCO_3_ MSs are obtained.

**Figure 1.**
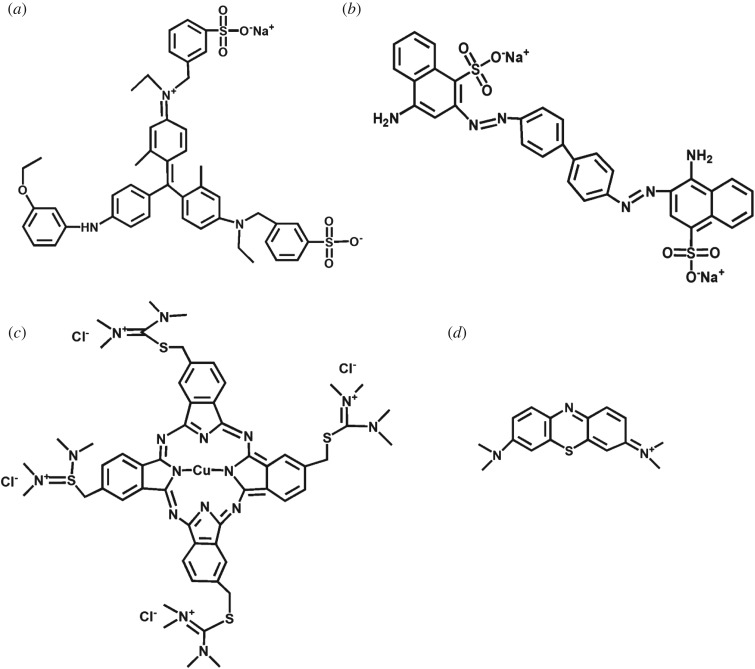
The chemical structures of the dyes used in the adsorption study. (*a*) Coomassie brilliant blue G-250; (*b*) Congo red; (*c*) Alcian blue; (*d*) methylene blue.

To investigate the adsorption properties of obtained core--shell structured CaCO_3_ MSs, as illustrated in figure 1, we chose Coomassie brilliant blue, Congo red, Alcian blue and methylene blue as model dyes. Calcination is used as a simple and effective desorption method, because calcium carbonate materials can remain stable, but dye molecules will be converted into carbon dioxide during calcination. We believe that such core–shell structured CaCO_3_ MSs can be considered as easily separated, high-efficiency and recyclable adsorbent for removal of dyes.

## 2. Experimental

### 2.1. Materials

Hesperidin and ammonia (28%, analytical reagent) were purchased from Sigma Chemical Co. (St Louis, MO, USA). CaCl_2_ (analytical reagent), NaHCO_3_ (analytical reagent) and NH_3_·H_2_O (analytical reagent) were purchased from Sinopharm Chemical Regent Co. Ltd (Shanghai, China). Coomassie brilliant blue G-250 (CBB), Congo red (CR), Alcian blue (AB) and methylene blue (MB) were purchased from Sigma Aldrich. Triply distilled deionized water was used during all the applications.

### 2.2. Preparation of CaCO_3_ microspheres

The core–shell structured CaCO_3_ MSs were synthesized by a facile, one-pot method. Firstly, 0.4% (wt %) Hesp solution was prepared by alkaline aqueous solution (pH = 11.0, adjusted by ammonia and HCl). Then, CaCl_2_ (5 ml, 1 mol l^−1^), NaHCO_3_ (10 ml, 1 mol l^−1^) and the prepared Hesp solution (5 ml) were added into a beaker (150 ml), into which 80 ml of water was further added. The mixture was vortex-stirred (IKA, Vortex, Genius 3) to obtain a homogeneous solution. After that, the solution was heated to 65°C and kept for 20 min. The beaker and 100 ml of ammonia (in another beaker) were placed in a closed desiccator for 24 h at room temperature. By NH_3_ diffusing and dissolving in the solution, the mineralization of CaCO_3_ MSs was initiated. The products were collected by centrifugation, and rinsed with deionized water several times. Finally, the obtained products were dried at 70°C for further analysis.

### 2.3. Characterization

The morphologies of the obtained CaCO_3_ MSs were investigated by scanning electron microscopy (SEM, Hitachi, S4800, Tokyo, Japan) and transmission electron microscopy (TEM, JEM-1200EX, Tokyo, Japan). The composition of the CaCO_3_ MSs was identified by using Fourier transform infrared (FTIR) spectroscopy (Shimadzu, Kyoto, Japan) in the range of 4000 to 400 cm^−1^ with the KBr disc method. The as-prepared CaCO_3_ MSs were examined with powder X-ray diffraction analysis (XRD, Shimadzu, Kyoto, Japan) with Cu Kα radiation to obtain the crystallographic structure of the MSs. N_2_ adsorption–desorption measurements were performed on a ASAP-2460 instrument with the MicroActive software (sample mass: 0.1269 g; equilibration interval: 10 s; sample density: 1.000 g cm^−3^), using Brunauer–Emmett–Teller (BET) calculations for surface area and Barrett–Joyner–Halenda calculations for pore size distribution.

The adsorption experiments of CBB, CR, AB and MB from an aqueous medium on the core–shell structured CaCO_3_ MSs were studied by a UV--visible absorption spectrophotometer (PerkinElmer Lambda 605S UV/Vis spectrometer). The dye solution was centrifuged; then, the supernatant was analysed to get absorbance by the UV--visible spectrometer at each maximum absorption wavelength (CBB (585 nm), CR (498 nm), AB (600 nm) and MB (664 nm)).

### 2.4. Adsorption experiments

The adsorption of CBB (500 mg l^−1^), CR (500 mg l^−1^), AB (500 mg l^−1^) and MB (200 mg l^−1^) from water by core–shell structured CaCO_3_ MSs can be described briefly as follows.

The effect of the adsorbent dosage (AD) on the adsorption process was investigated by adding different dosages of CaCO_3_ MSs, which varied in the ranges of 0–50 mg (CBB), 0–50 mg (CR), 0–200 mg (AB) and 0–300 mg (MB) for a 10 ml sample bottle containing 4 ml of dye solution. After a given adsorption time, the mixture was centrifuged, and then the supernatant was analysed to obtain the absorbance with the UV--visible spectrometer at each maximum absorption wavelength.

According to the experimental results listed in this paper, the adsorption efficiency of CaCO_3_ MSs for anionic dyes (CBB and CR) has already reached approximately 100% when the AD increases to 20 mg. Therefore, the adsorbent dosage of 20 mg (for CBB and CR) was used to determine the adsorption time. To determine the adsorption time of AB and MB, the adsorbent dosage was set as 100 mg and 200 mg , respectively.

CaCO_3_ cubic microcrystals were prepared and used as dye adsorbent in the contrast experiments.

To investigate the adsorption efficiency in the above experiments, we adopt a similar calibration curve method based on UV--visible spectroscopy as reported in our previous work [24,25].

The equilibrium adsorption capacity (*q*_e_) was calculated according to the following equation:

2.1 $$q_{e}=\frac{(C_{0}-C_{e})V}{m},$$

where *C*_0_ and *C*_e_ are initial and equilibrium dye concentrations, respectively (mg l^−1^), *V* is the solution volume (l) and *m* is the adsorbent dosage of CaCO_3_ MSs (g).

### 2.5. Recycling experiment

The recyclability of the adsorbent is very important. After dye adsorption, the CaCO_3_ MSs were separated (from the dye solution), dried and burned at 450°C for 2 h to desorb the dyes by using a muffle furnace. Then, those calcinated CaCO_3_ MSs were reused to adsorb dyes according to the previous experiment steps. To investigate the recyclability of those core–shell structured CaCO_3_ MSs, such an ‘adsorption--calcination (desorption)--adsorption’ cycle was carried out five times in each group.

## 3. Results and discussion

**Figure 2.**
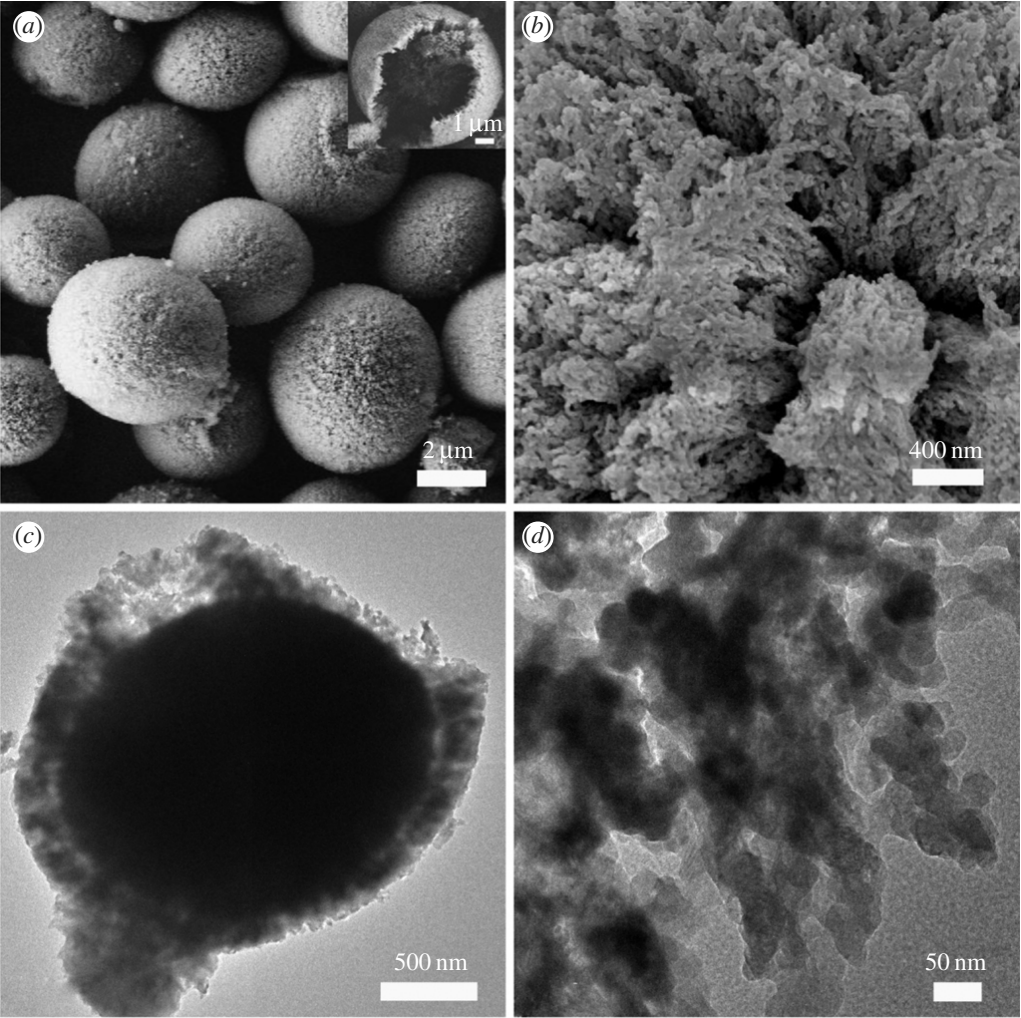
SEM and TEM images. (*a*) SEM image of the obtained CaCO_3_ microspheres. (*b*) SEM image of the obtained CaCO_3_ microspheres sprayed with gold. (*c*) TEM image of the obtained CaCO_3_ microspheres. (*d*) A high-resolution TEM image of the edge of the microsphere.

The SEM image (figure 2*a*) clearly shows that the obtained CaCO_3_ crystals are MSs (3–5 µm in diameter) with rough surfaces. The inset image in figure 2*a* shows a single CaCO_3_ MS with a broken surface. It is obvious that this CaCO_3_ MS has a thin shell which surrounds the inner core. The detailed surface features of the CaCO_3_ MS are shown in figure 2*b*. It is clear that the porous surface of such a MS is composed of units of numerous small particles. The TEM image in figure 2*c* further confirms the core–shell structure of the obtained products. It clearly shows that the CaCO_3_ MS has a thin, porous and loose shell, and a condensed core. The magnified TEM image (figure 2*d*) provides the detailed structure of the shell shown in figure 2*c*. It is the aggregate of many spherical nanocrystals with an average size of approximately 30 nm in diameter. Owing to the hierarchical core–shell structure, these CaCO_3_ MSs are endowed with a large surface area. Thus, BET measurements were carried out to study the porosity and pore size distribution of the MS samples. A BET surface area of 211.77 m^2^ g^−1^ is achieved for CaCO_3_ MSs with an average pore size of 7.8 nm (electronic supplementary material, figure S1). These data suggest that the CaCO_3_ MSs can offer lots of adsorption sites for organic dye molecules.

**Figure 3.**
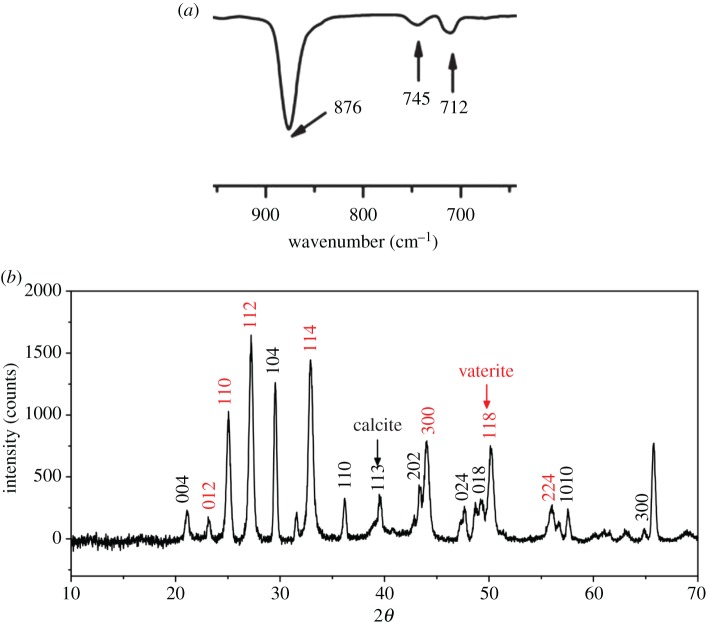
FTIR spectrum (*a*) and XRD pattern (*b*) of CaCO_3_ microspheres.

The absorption peaks (FTIR spectrum, figure 3*a*) located at 712, 876 cm^−1^ (ascribed to characteristic absorption peaks of calcite) and 745 cm^−1^ (ascribed to characteristic absorption peaks of vaterite) indicate that these core–shell structured CaCO_3_ MSs are composed of both calcite and vaterite [24]. The XRD pattern in figure 3*b* further proved the polycrystalline property of the CaCO_3_ MSs. The reflections at 2θ = 21.1, 29.6, 36.0, 39.5, 43.3, 47.2, 47.8 48.6 and 65.2° are attributable to planes of calcite (JCPDS card no. 83-0578), while reflections at 23.1, 25.1, 27.3, 32.9, 43.9, 50.3 and 55.8° are attributable to planes of vaterite (JCPDS card no. 72-0506). Rao's equation is adopted to calculate the relative fractions of vaterite (*f*_v_) and calcite in the crystalline phase according to the literature [26,27]:

3.1 $$f_{v}=\frac{I_{110V}+I_{112V}+I_{114V}}{I_{110V}+I_{112V}+I_{114V}+I_{104C}}.$$

The result indicates that the calcite and vaterite contents in the core–shell structured CaCO_3_ microspheres are 76.01% and 23.99%, respectively.

The prepared CaCO_3_ MSs were rinsed thoroughly. However, Hesp was still found in the obtained CaCO_3_ MSs. We used thermogravimetric analysis to determine the content of Hesp in the obtained core–shell structured CaCO_3_ MSs. The result in electronic supplementary material, figure S2, confirms that the content of Hesp in CaCO_3_ MSs is less than 1 wt%. Therefore, the influence of Hesp on the adsorption performance of CaCO_3_ might be negligible.

**Figure 4.**
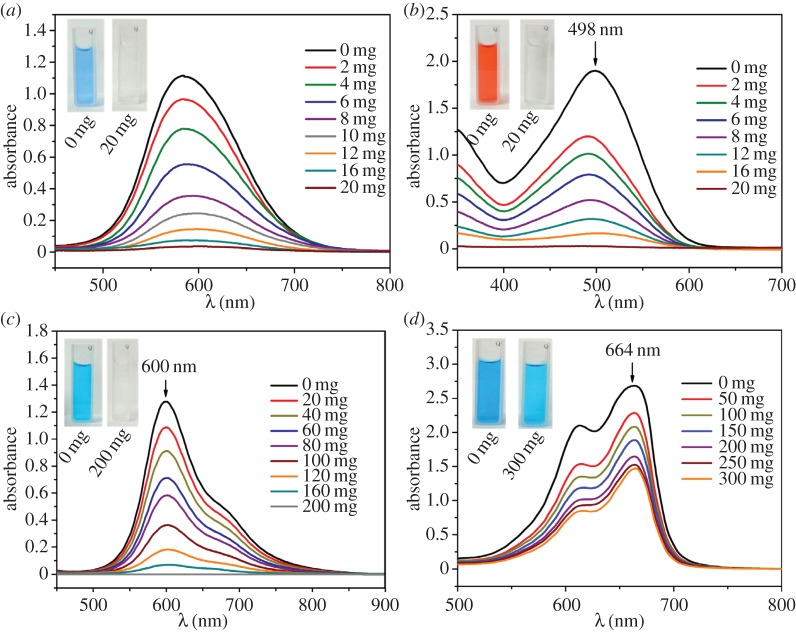
The UV--visible absorption spectra at different adsorbent dosages of the obtained CaCO_3_ microspheres for different dyes. Inset shows photographs of dye solution before (left) and after (right) adsorption on CaCO_3_ microspheres. (*a*) Coomassie brilliant blue G-250; (*b*) Congo red; (*c*) Alcian blue; (*d*) methylene blue.

Figure 4 shows the effect of the AD on the adsorption capability of CaCO_3_ MSs in the solution of the four dyes (CBB, CR, AB and MB). Figure 4*a* shows that the increase in the AD of CaCO_3_ MSs will decrease the absorbance at 585 nm (maximum absorption peak of CBB) gradually. Moreover, these absorption curves tend to be more flat on increasing the AD to 10 mg. When the AD reaches 20 mg, it can be found that nearly all (99.57 wt%) the CBB dye has successfully been adsorbed by the CaCO_3_ MSs. The insets in figure 4*a* show the images of the CBB solution (500 mg l^−1^, left image) and the supernatant after adsorption by 20 mg of CaCO_3_ MSs (right image). It is obvious that the blue colour of the CBB solution has faded in the supernatant. Both the disappearance of the absorption peak and the fading of the colour indicate that the core–shell structured CaCO_3_ MSs have strong adsorption capability for CBB. From the adsorption curves and insets shown in figure 4*b*, it can be found that the adsorption of CR by the CaCO_3_ MSs is similar to that of CBB in figure 4*a*. When the AD reaches 20 mg, the added CaCO_3_ MSs can strongly adsorb almost all the CR dye (99.60 wt%) from the solution. Figure 4*c* indicates that the CaCO_3_ MSs present similar adsorption behaviour in AB solution as in CBB and CR solutions. The difference is that a larger amount of CaCO_3_ MSs (200 mg) than 50 mg is needed to adsorb 99.79 wt% of AB from the solution. However, the adsorption of CaCO_3_ in the MB solution is much different from that of the other three dyes (figure 4*d*). By increasing the added amount of CaCO_3_ MSs from 0 to 200 mg, the absorbance at 664 nm (maximum absorption peak of MB) is decreased gradually. However, when the adsorbent dosage is further increased from 250 mg to 300 mg, such decrease in absorbance is not as obvious as before. The inset in figure 4*d* indicates that the blue colour of the MB solution has not faded thoroughly in the supernatant after being adsorbed by 300 mg of CaCO_3_ MSs; 54.83 wt% of MB molecules are still left in the supernatant.

**Figure 5.**
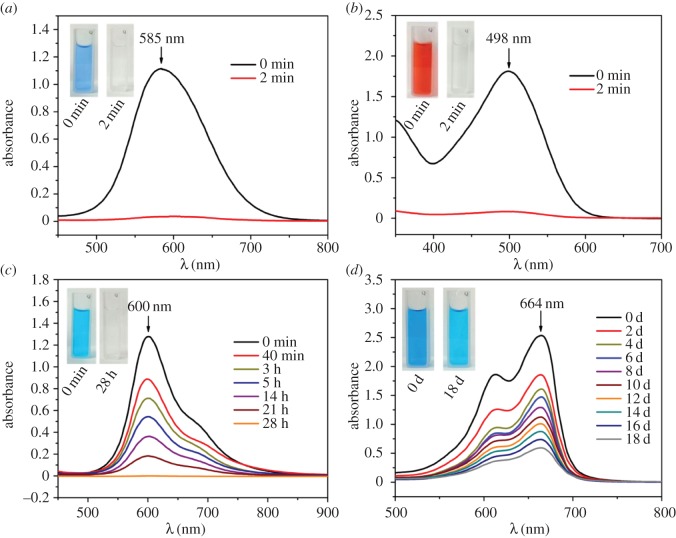
The UV--visible absorption spectra at different adsorption times on the obtained CaCO_3_ microspheres for different dyes. Inset shows photographs of dye solution before (left) and after (right) adsorption on CaCO_3_ microspheres. (*a*) Coomassie brilliant blue G-250; (*b*) Congo red; (*c*) Alcian blue; (*d*) methylene blue.

The above results (figure 4) demonstrate that the obtained CaCO_3_ MSs present different adsorption capacities for CBB, CR, AB and MB under optimal adsorbent dosages. Figure 5 further provides the effect of adsorption time (AT) on the adsorption behaviour of CaCO_3_ MSs in the solutions of the four dyes. It is obvious that CaCO_3_ MSs can completely remove CBB or CR molecules very rapidly (in 2 min, figure 5*a*,*b*). For AB, CaCO_3_ MSs need a long time (28 h, figure 5*c*) to adsorb almost all dye molecules. Figure 5*d* reveals that CaCO_3_ MSs can remove about 76.61 wt % MB from water after a quite long AT of 18 days.

The results in figures 4 and 5 prove that the obtained CaCO_3_ MSs can efficiently adsorb both CBB and CR as quickly as 2 min. For AB molecules, CaCO_3_ MSs can also completely remove them, but the AT is 28 h. For MB, CaCO_3_ MSs cannot efficiently remove all dye molecules, even with a given AT of more than half a month. The above difference may be ascribed to the chemical structure of the four dyes. As illustrated in figure 1, both CBB and CR are anionic dyes with sulfonate groups, which can form a complex with the calcium ions of CaCO_3_ MSs. This feature ensures that CBB and CR have a strong combination with the CaCO_3_ MSs. Thus, CaCO_3_ MSs can adsorb CBB and CR efficiently and rapidly. With regard to AB and MB (figure 1*c*,*d*), they are cationic dyes with tertiary ammonium and quaternary ammonium groups. Those tertiary ammonium groups will bind the Ca^2+^ of the CaCO_3_ MSs. However, positively charged quaternary ammonium groups will repel the Ca^2+^ on the CaCO_3_ MSs. For AB, it has more tertiary ammonium groups (12) than quaternary ammonium groups (4). Thus, CaCO_3_ MSs can efficiently adsorb AB molecules due to the tertiary ammonium groups. The AT is quite long (28 h) because of the exclusion between quaternary ammonium groups and the Ca^2+^ ions. With regard to MB, its molecular size is much smaller than AB, so the exclusion between quaternary ammonium groups and the Ca^2+^ ions is stronger than that of AB. Therefore, the CaCO_3_ MSs only remove about 76.61 wt% MB from water after a quite long AT of 18 days.

**Figure 6.**
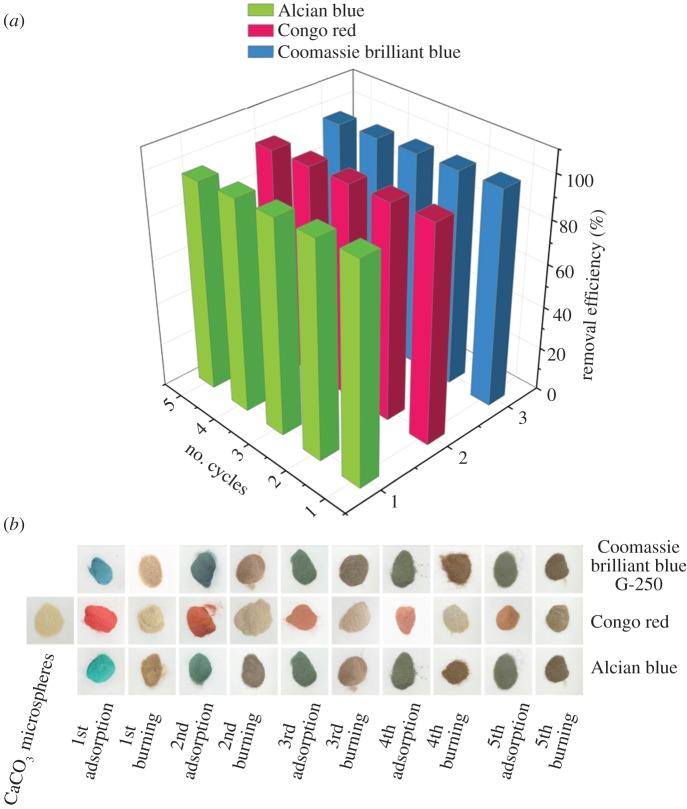
The dye adsorption efficiency on the obtained CaCO_3_ microspheres in five successive cycles of desorption–adsorption. (*a*) Three-dimensional histogram and (*b*) corresponding photos of the CaCO_3_ microspheres before and after adsorption or calcination.

The recycling experiments were performed for five cycles (adsorption–desorption) and the results are depicted in figure 6. In the first run, very high removal efficiencies are seen for CBB, CR and AB of up to 99.57%, 99.60% and 99.79%, respectively. Moreover, it was found that 96.66% of CBB, 97.23% of CR and 96.41% of AB was still adsorbed after the 5th cycle (figure 6*a*). Figure 6*b* further directly shows the adsorption and desorption of dyes by the CaCO_3_ MSs in the recycling experiments. According to the SEM image (electronic supplementary material, figure S3) and BET data (electronic supplementary material, figure S4) of these CaCO_3_ MSs, which have been used for five recycling experiments, it is clear that these CaCO_3_ MSs maintain their original structure well after five recycling experiments. This indicates the obtained core–shell CaCO_3_ MSs are stable in the recycling experiments. Electronic supplementary material, figure S4, shows that the BET surface area of CaCO_3_ MSs after five cycles has no obvious change.

Furthermore, it was found that the removal efficiency of dyes has declined slightly to 93.86% for CBB, 95.63% for CR and 92.40% for AB after the 10th cycle. This suggests that the obtained CaCO_3_ MSs are stable in the recycling experiments and maintain a high removal efficiency for dyes.

**Figure 7.**
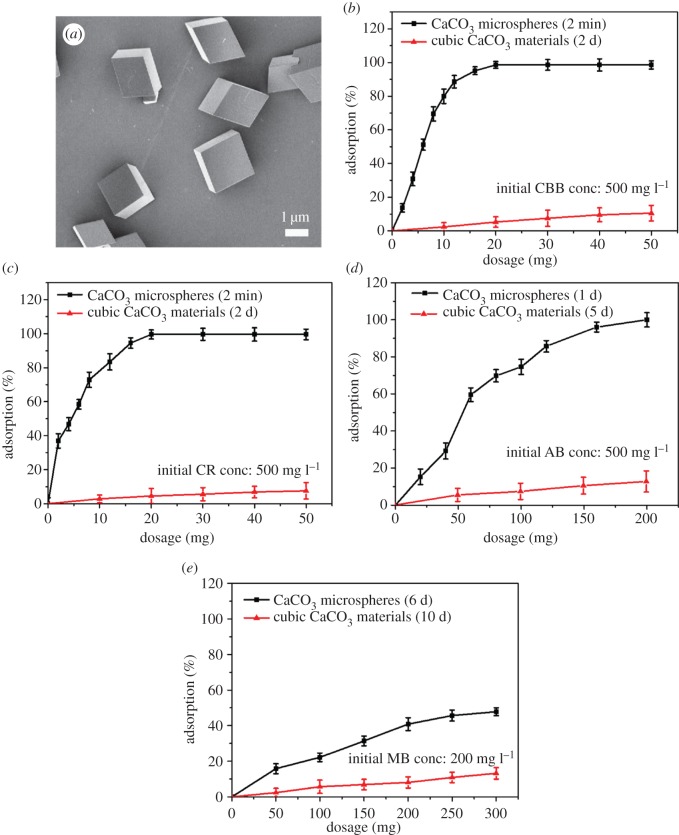
Comparison of the dye adsorption efficiency on the obtained CaCO_3_ microspheres and cubic CaCO_3_ materials for different dyes. (*a*) SEM image of the cubic CaCO_3_ materials; (*b*) Coomassie brilliant blue G-250B; (*c*) Congo red; (*d*) Alcian blue; (*e*) methylene blue.

We use calcite microcrystals as adsorbent in the contrast experiment of dye adsorption (figure 7). The SEM image (figure 7*a*) reveals that the morphology of the calcite materials is that of smooth cubic microcrystals. The adsorption of dyes by CaCO_3_ materials could be attributed to the electrostatic interaction between Ca^2+^ and organic dye molecules. Compared to the smooth, cubic calcite microcrystals, the core–shell structured CaCO_3_ MSs have a much larger surface area which can provide more adsorption sites (Ca^2+^). Therefore, the removal of dyes by core–shell CaCO_3_ MSs from aqueous solution is a more rapid and efficient process (figure 7*b*–*e*) than that by using cubic CaCO_3_ materials as adsorbents.

A comparison of the adsorption capacity (*q*_e_) for Congo red in this paper and reported results is presented in table 1. It is obvious that the adsorption capacity of Congo red by the presented core–shell structured CaCO_3_ MSs is larger (99.6 mg g^−1^) and the adsorption time is remarkably faster (2 min) than for those materials reported previously [28–32]. For instance, Sayğılı reported that the adsorption capacity of a biomagnetic composite as adsorbent for the removal of Congo red is 86.96 mg g^−1^, and the adsorption time for the equilibrium is 200 min [28]. Yu *et al.* reported that the adsorption capacity of mesoporous Fe_2_O_3_ is 53 mg g^−1^, and the adsorption time for the equilibrium is 120 min [29]. Therefore, these core–shell structured CaCO_3_ MSs can be used as a rapid and efficient adsorbent to remove anionic dyes from aqueous solution.

**Table 1.** Adsorption capacity (*q*_e_) and adsorption time for Congo red in the literature and in this study.

adsorbent	adsorption capacity (mg g^−1^)	adsorption efficiency (%)	adsorption time (min)	reference
biomagnetic composite	86.96	equilibrium	200	[28]
mesoporous Fe_2_O_3_	53	equilibrium	120	[29]
supramolecular adsorbent	70.1	equilibrium	120	[30]
chemically modified lignocellulosic jute fibre	27.12	99.87	15–30	[31]
vaterite CaCO_3_	32.60	79.93	180	[32]
core–shell structured CaCO_3_ microspheres	99.6	99.6	2	this work

## 4. Conclusion

In this work, we prepared core–shell structured CaCO_3_ MSs by a facile, one-pot method at room temperature. The results suggest that these CaCO_3_ MSs can rapidly and efficiently remove 99–100% of CBB and CR dyes within 2 min. We also found that these core–shell structured CaCO_3_ MSs have a high recycling capability for removing dyes from water. Our results demonstrate that the obtained core–shell structured CaCO_3_ MSs can be used as an ideal, rapid, efficient and recyclable adsorbent to remove dyes from aqueous solution.

## Supplementary Material

Figures S1 - S5

## Data accessibility

We include all the experimental data in the electronic supplementary material, which are available at http://dx.doi.org/10.5061/dryad.m1b34 [33].

## References

[1] Yao L, Zhang L, Wang R, Chou S, Dong Z 2016 A new integrated approach for dye removal from wastewater by polyoxometalates functionalized membranes. J. Hazard. Mater. 301, 462–470. (doi:10.1016/j.jhazmat.2015.09.027) 26410275 10.1016/j.jhazmat.2015.09.027

[2] Zhu X, Ni J, Wei J, Xing X, Li H 2011 Destination of organic pollutants during electrochemical oxidation of biologically-pretreated dye wastewater using boron-doped diamond anode. J. Hazard. Mater. 189, 127–133. (doi:10.1016/j.jhazmat.2011.02.008) 21377794 10.1016/j.jhazmat.2011.02.008

[3] Pourjavadi A, Nazari M, Kabiri B, Hosseini SH, Bennett C 2016 Preparation of porous graphene oxide/hydrogel nanocomposites and their ability for efficient adsorption of MB. RSC Adv. 6, 10 430–10 437. (doi:10.1039/C5RA21629J)

[4] Madrakian T, Afkhami A, Mahmood-Kashani H, Ahmadi M 2013 Adsorption of some cationic and anionic dyes on magnetite nanoparticles-modified activated carbon from aqueous solutions: equilibrium and kinetics study. J. Iran. Chem. Soc. 10, 481–489. (doi:10.1007/s13738-012-0182-4)

[5] Sun L, Wan S, Luo W 2013 Biochars prepared from anaerobic digestion residue, palm bark, and eucalyptus for adsorption of cationic MB dye: characterization, equilibrium, and kinetic studies. Bioresour. Technol. 140, 406–413. (doi:10.1016/j.biortech.2013.04.116) 23714096 10.1016/j.biortech.2013.04.116

[6] Liu Q, Wang L, Xiao A, Gao J, Ding W, Yu H, Ericson M 2010 Templated preparation of porous magnetic microspheres and their application in removal of cationic dyes from wastewater. J. Hazard. Mater. 181, 586–592. (doi:10.1016/j.jhazmat.2010.05.053) 20619794 10.1016/j.jhazmat.2010.05.053

[7] Koley P, Sakurai M, Takei T, Aono M 2016 Facile fabrication of silk protein sericin-mediated hierarchical hydroxyapatite-based bio-hybrid architectures: excellent adsorption of toxic heavy metals and hazardous dye from wastewater. RSC Adv. 6, 86 607–86 616. (doi:10.1039/C6RA12818A)

[8] Ahmad A, Mohd-Setapar SH, Chuong CS, Khatoon A, Wani WA, Kumar R 2015 Recent advances in new generation dye removal technologies: novel search for approaches to reprocess wastewater. RSC Adv. 5, 30 801–30 818. (doi:10.1039/C4RA16959J)

[9] Fan Y, Liu HJ, Zhang Y, Chen Y 2015 Adsorption of anionic MO or cationic MB from MO/MB mixture using polyacrylonitrile fiber hydrothermally treated with hyperbranched polyethylenimine. J. Hazard. Mater. 283, 321–328. (doi:10.1016/j.jhazmat.2014.09.042) 25305362 10.1016/j.jhazmat.2014.09.042

[10] Rafatullah M, Sulaiman O, Hashim R, Ahmad A 2010 Adsorption of methylene blue on low-cost adsorbents: a review. J. Hazard. Mater. 177, 70–80. (doi:10.1016/j.jhazmat.2009.12.047) 20044207 10.1016/j.jhazmat.2009.12.047

[11] Vakili M, Rafatullah M, Salamatinia B, Abdullah AZ, Ibrahim MH, Tan KB, Amouzgar P 2014 Application of chitosan and its derivatives as adsorbents for dye removal from water and wastewater: a review. Carbohydr. Polym. 113, 115–130. (doi:10.1016/j.carbpol.2014.07.007) 25256466 10.1016/j.carbpol.2014.07.007

[12] Hassan SS, Awwad NS, Aboterika AH 2009 Removal of synthetic reactive dyes from textile wastewater by Sorel's cement. J. Hazard. Mater. 162, 994–999. (doi:10.1016/j.jhazmat.2008.05.138) 18635316 10.1016/j.jhazmat.2008.05.138

[13] Mishra A, Bajpai M 2005 Flocculation behaviour of model textile wastewater treated with a food grade polysaccharide. J. Hazard. Mater. 118, 213–217. (doi:10.1016/j.jhazmat.2004.11.003) 15721546 10.1016/j.jhazmat.2004.11.003

[14] Hu ZJ, Xiao Y, Zhao DH, Shen YL, Gao HW 2010 Preparation of dye waste-barium sulfate hybrid adsorbent and application in organic wastewater treatment. J. Hazard. Mater. 175, 179–186. (doi:10.1016/j.jhazmat.2009.09.146) 19850409 10.1016/j.jhazmat.2009.09.146

[15] Dong S, Cui Y, Wang Y, Li Y, Hu L, Sun J 2014 Designing three-dimensional acicular sheaf shaped BiVO_4_/reduced graphene oxide composites for efficient sunlight-driven photocatalytic degradation of dye wastewater. Chem. Eng. J. 249, 102–110. (doi:10.1016/j.cej.2014.03.071)

[16] Bhatt AS, Sakaria PL, Vasudevan M, Pawar RR, Sudheesh N, Bajaj HC, Mody HM 2012 Adsorption of an anionic dye from aqueous medium by organoclays: equilibrium modeling, kinetic and thermodynamic exploration. RSC Adv. 2, 8663–8671. (doi:10.1039/c2ra20347b)

[17] Goel NK, Kumar V, Pahan S, Bhardwaj YK, Sabharwal S 2011 Development of adsorbent from Teflon waste by radiation induced grafting: equilibrium and kinetic adsorption of dyes. J. Hazard. Mater. 193, 17–26. (doi:10.1016/j.jhazmat.2011.05.026) 21855214 10.1016/j.jhazmat.2011.05.026

[18] Fan L, Luo C, Sun M, Qiu H, Li X 2013 Synthesis of magnetic β-cyclodextrin–chitosan/graphene oxide as nanoadsorbent and its application in dye adsorption and removal. Colloids Surf. B Biointerfaces 103, 601–607. (doi:10.1016/j.colsurfb.2012.11.023) 23261586 10.1016/j.colsurfb.2012.11.023

[19] Patra AS, Ghorai S, Ghosh S, Mandal B, Pal S 2016 Selective removal of toxic anionic dyes using a novel nanocomposite derived from cationically modified guar gum and silica nanoparticles. J. Hazard. Mater. 301, 127–136. (doi:10.1016/j.jhazmat.2015.08.042) 26348145 10.1016/j.jhazmat.2015.08.042

[20] Zhang J, Yao B, Ping H, Fu Z, Li Y, Wang W, Zhang F 2016 Template-free synthesis of hierarchical porous calcium carbonate microspheres for efficient water treatment. RSC Adv. 6, 472–480. (doi:10.1039/C5RA18366A)

[21] Ai L, Zeng Y 2013 Hierarchical porous NiO architectures as highly recyclable adsorbents for effective removal of organic dye from aqueous solution. Chem. Eng. J. 215, 269–278. (doi:10.1016/j.cej.2012.10.059)

[22] Wu L, Fang S, Ge L, Han C, Qiu P, Xin Y 2015 Facile synthesis of Ag@CeO_2_ core–shell plasmonic photocatalysts with enhanced visible-light photocatalytic performance. J. Hazard. Mater. 300, 93–103. (doi:10.1016/j.jhazmat.2015.06.062) 26163484 10.1016/j.jhazmat.2015.06.062

[23] Xiao G, Su H, Tan T 2015 Synthesis of core–shell bioaffinity chitosan–TiO_2_ composite and its environmental applications. J. Hazard. Mater. 283, 888–896. (doi:10.1016/j.jhazmat.2014.10.047) 25464333 10.1016/j.jhazmat.2014.10.047

[24] Yang T, Wan Z, Liu Z, Li H, Wang H, Lu N, Chen Z, Mei X, Ren X 2016 *In situ* mineralization of anticancer drug into calcium carbonate monodisperse nanospheres and their pH-responsive release property. Mater. Sci. Eng. C 63, 384–392. (doi:10.1016/j.msec.2016.03.009) 10.1016/j.msec.2016.03.009 27040233

[25] Chen ZH, Wang CH, Chen JZ, Li XD 2013 Biocompatible, functional spheres based on oxidative coupling assembly of green tea polyphenols. J. Am. Chem. Soc. 135, 4179–4182. (doi:10.1021/ja311374b) 23470166 10.1021/ja311374b

[26] Rao MS 1973 Kinetics and mechanism of the transformation of vaterite to calcite. Bull. Chem. Soc. Jpn 46, 1414–1417. (doi:10.1246/bcsj.46.1414)

[27] Wang A, Yang Y, Zhang X, Liu X, Cui W, Li J 2016 Gelatin-assisted synthesis of vaterite nanoparticles with higher surface area and porosity as anticancer drug containers *in vitro*. ChemPlusChem 81, 194–201. (doi:10.1002/cplu.201500515) 10.1002/cplu.201500515 31968759

[28] Sayğılı GA 2015 Synthesis, characterization and adsorption properties of a novel biomagnetic composite for the removal of Congo red from aqueous medium. J. Mol. Liq. 211, 515–526. (doi:10.1016/j.molliq.2015.07.048)

[29] Yu C, Dong X, Guo L, Li J, Qin F, Zhang L, Yan D 2008 Template-free preparation of mesoporous Fe_2_O_3_ and its application as absorbents. J. Phys. Chem. C 112, 13 378–13 382. (doi:10.1021/jp8044466)

[30] Chen M, Ding W, Wang J, Diao G 2013 Removal of azo dyes from water by combined techniques of adsorption, desorption, and electrolysis based on a supramolecular sorbent. Ind. Eng. Chem. Res. 52, 2403–2411. (doi:10.1021/ie300916d)

[31] Roy A, Adhikari B, Majumder SB 2013 Equilibrium, kinetic, and thermodynamic studies of azo dye adsorption from aqueous solution by chemically modified lignocellulosic jute fiber. Ind. Eng. Chem. Res. 52, 6502–6512. (doi:10.1021/ie400236s)

[32] Chong KY, Chia CH, Zakaria S, Sajab MS 2014 Vaterite calcium carbonate for the adsorption of Congo red from aqueous solutions. J. Environ. Chem. Eng. 2, 2156–2161. (doi:10.1016/j.jece.2014.09.017)

[33] Zhao M, Chen Z, Lv X, Zhou K, Zhang J, Tian X, Ren X, Mei X 2017 Data from: Preparation of Core-Shell Structured CaCO_3_ microspheres as ultra-fast and recyclable adsorbent for anionic dyes Dryad Digital Repository. (doi:10.5061/dryad.m1b34) 10.1098/rsos.170697 PMC5627111 28989771

