# Supplementary material for: Preparation of core–shell structured CaCO3 microspheres as rapid and recyclable adsorbent for anionic dyes
Source: R Soc Open Sci. 2017 Sep 6;4(9):170697. doi: 10.1098/rsos.170697 (PMC5627111; doi:10.1098/rsos.170697)
Supplement: Figures S1 - S5 [file rsos170697supp1.doc]

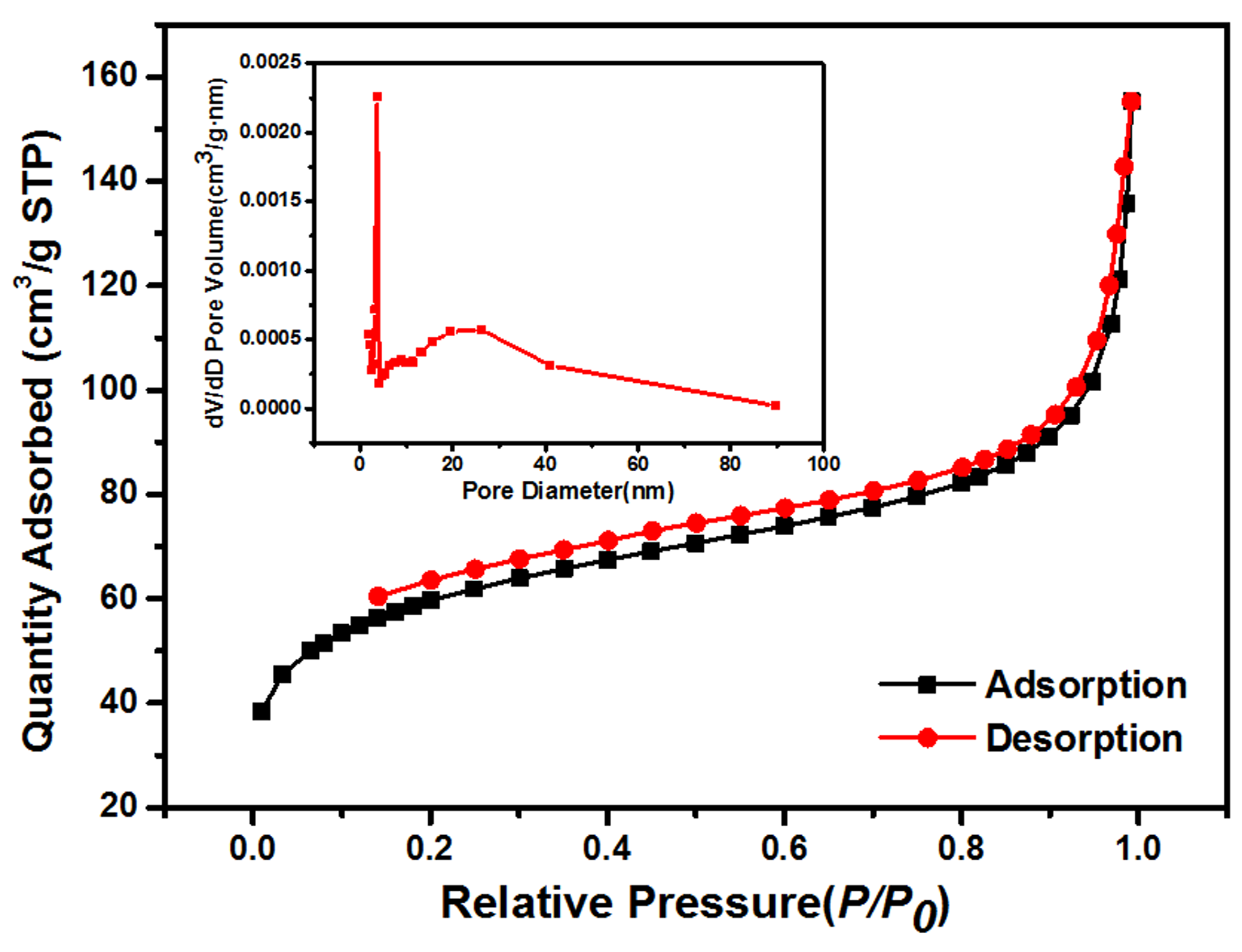


**Figure S1** N_2_ adsorption/desorption isotherms and pore-size distribution (inset) of the obtained core-shell structured CaCO_3_ MSs.


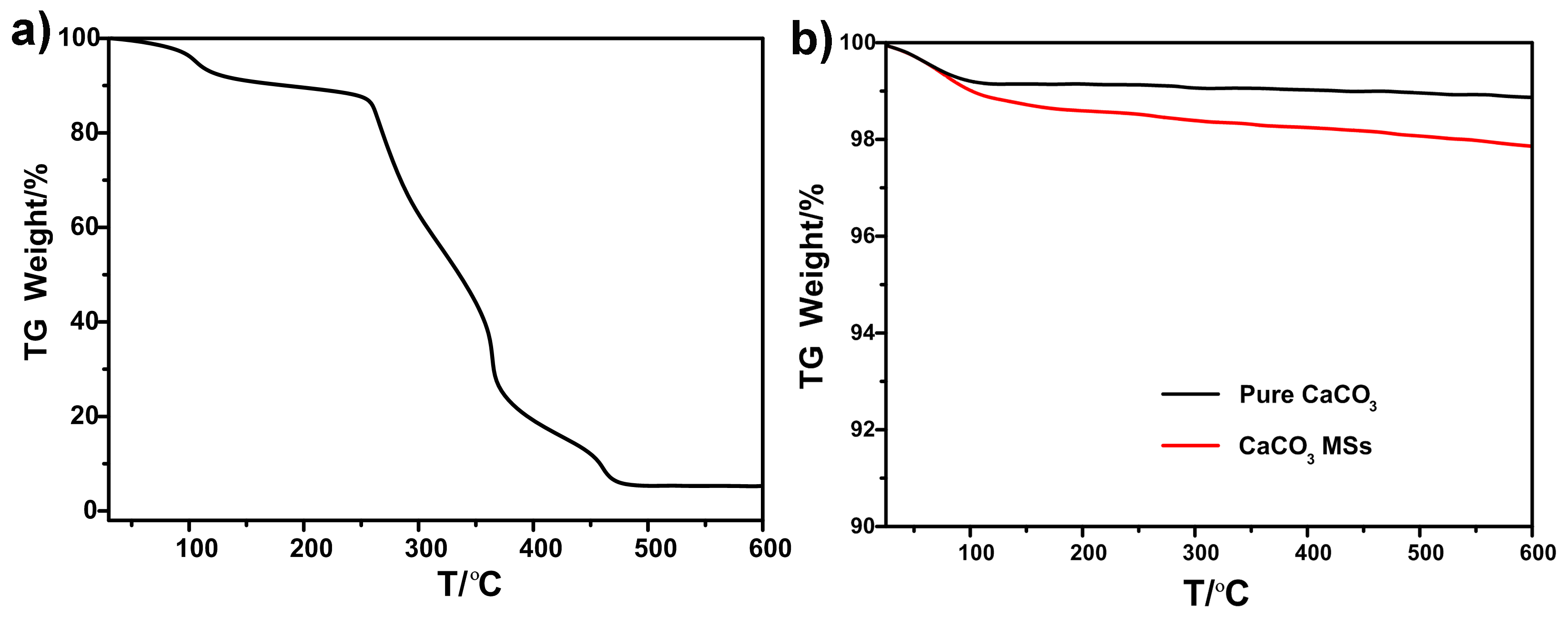


**Figure S2** TG analysis of Hesp (a), pure CaCO_3_ without Hesp and the obtained core-shell structured CaCO_3_ MSs (b).


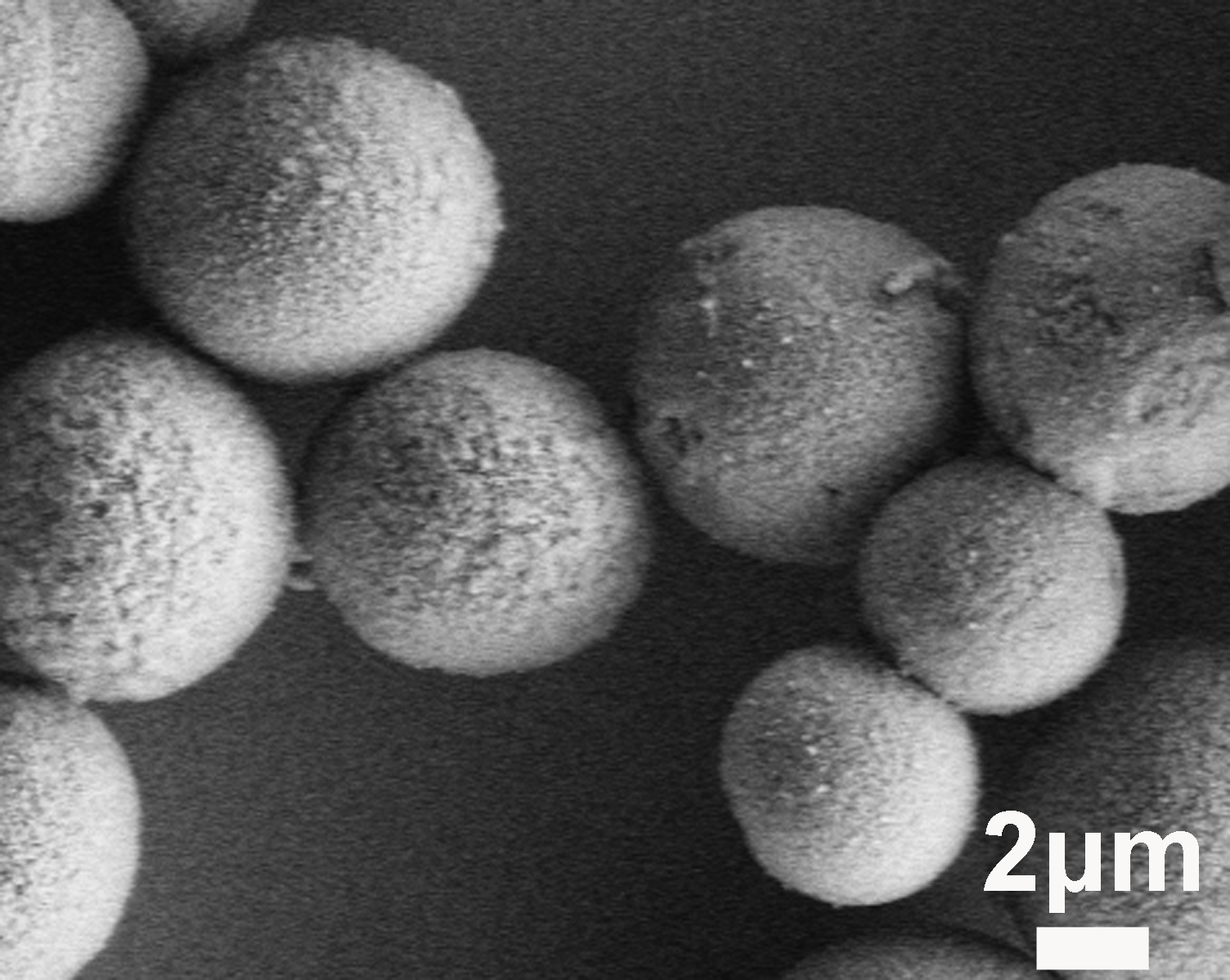


**Figure S3** SEM image of the CaCO_3_ MSs after 5 recycling experiments.


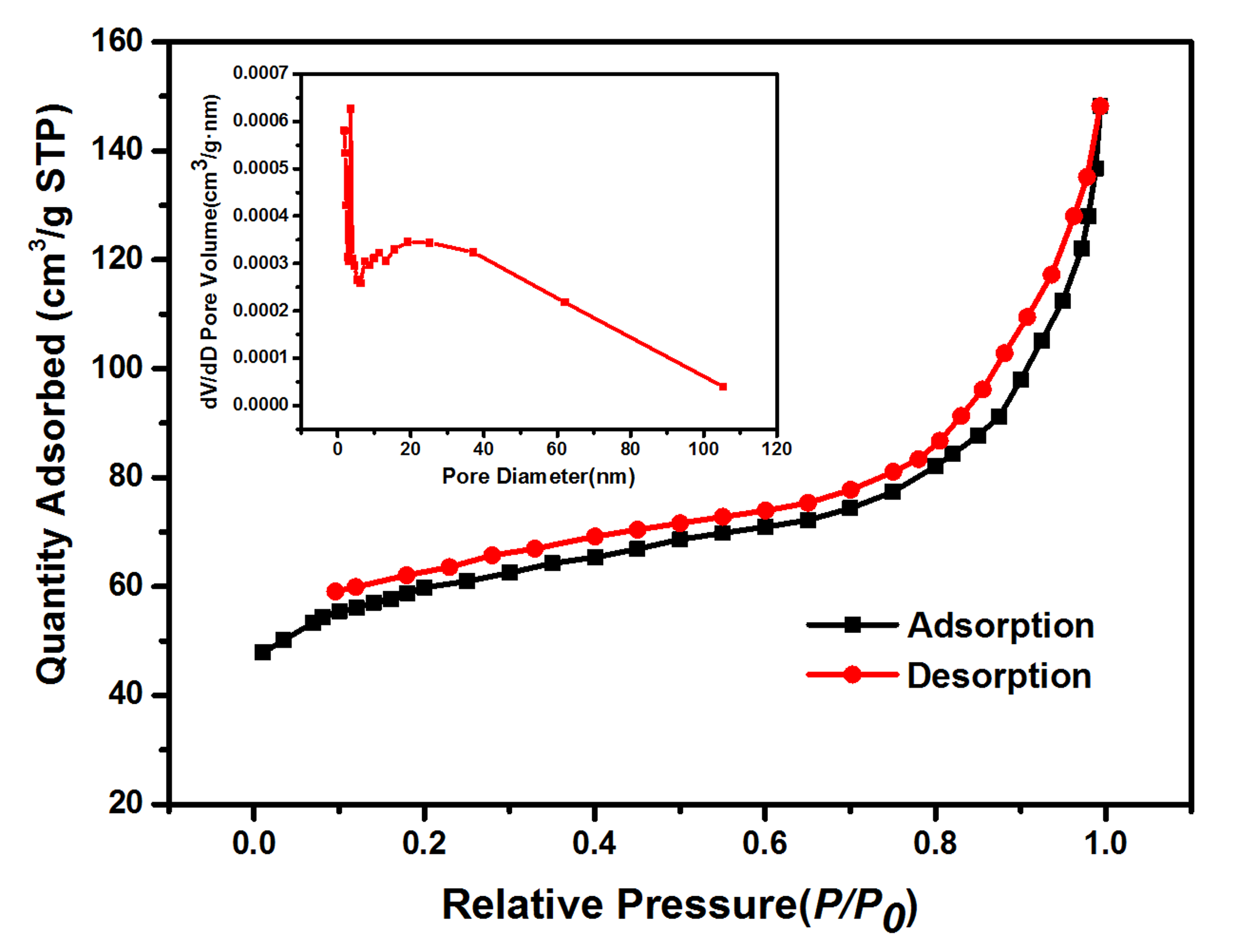


**Figure S4** N_2_ adsorption/desorption isotherms and pore-size distribution (inset) of the CaCO_3_ MSs after 5 recycling experiments.


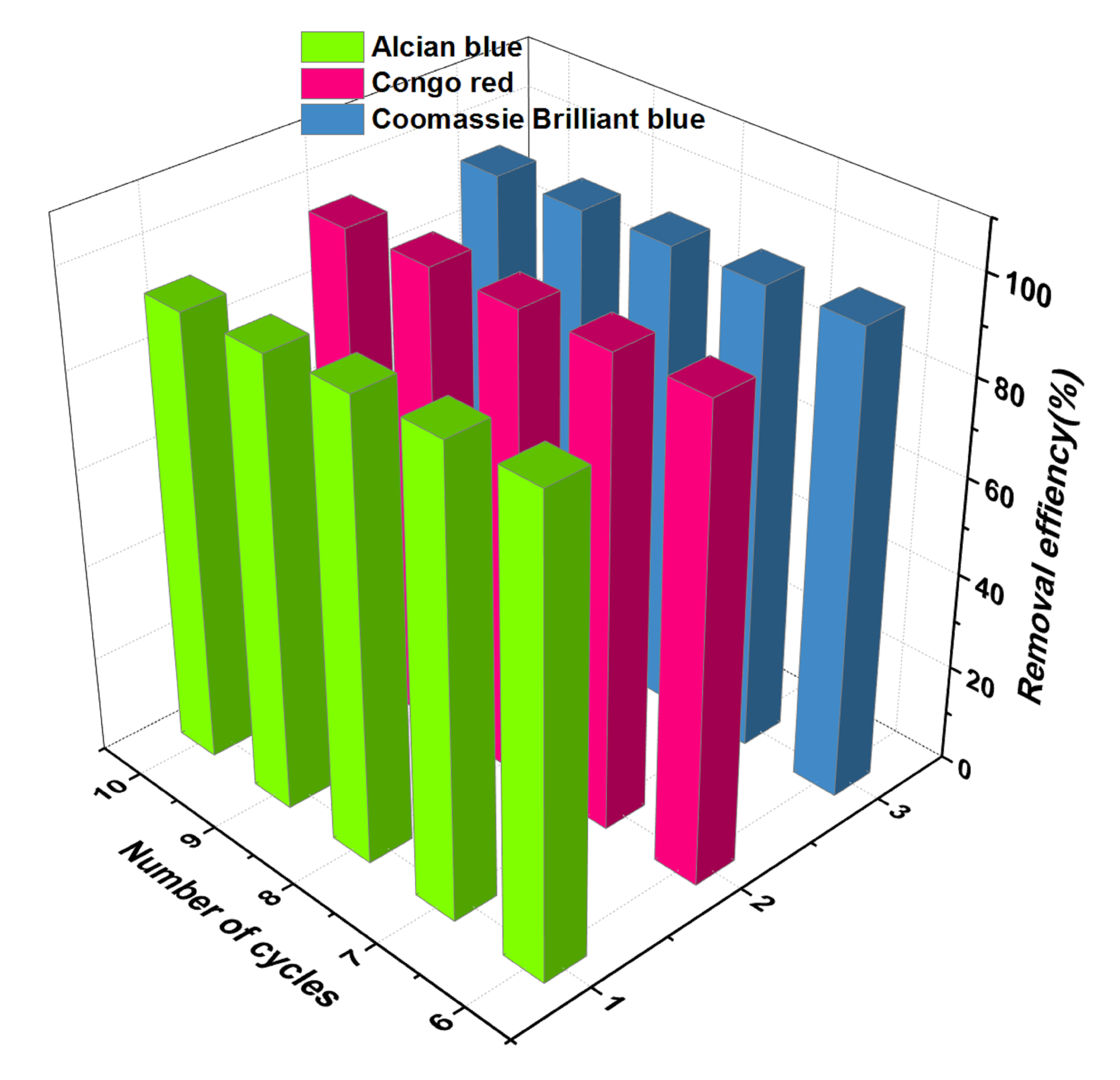


**Figure S5** Three-dimensional histogram of the dye adsorption efficiency on the CaCO_3_ MSs in 6-10th cycles of the adsorption-desorption.
